# Molecular Mechanisms of Skewed X-Chromosome Inactivation in Female Hemophilia Patients—Lessons from Wide Genome Analyses

**DOI:** 10.3390/ijms22169074

**Published:** 2021-08-23

**Authors:** Rima Dardik, Einat Avishai, Shadan Lalezari, Assaf A. Barg, Sarina Levy-Mendelovich, Ivan Budnik, Ortal Barel, Yulia Khavkin, Gili Kenet, Tami Livnat

**Affiliations:** 1 National Hemophilia Center, Sheba Medical Center, Ramat Gan 52621, Israel; rima.dardik@sheba.health.gov.il (R.D.); einat.avishai@sheba.health.gov.il (E.A.); shadan.lalezari@sheba.health.gov.il (S.L.); assaf.barg@sheba.health.gov.il (A.A.B.); Sarina.Levy@sheba.health.gov.il (S.L.-M.); Gili.Kenet@sheba.health.gov.il (G.K.); 2 Amalia Biron Research Institute of Thrombosis and Hemostasis, Sackler School of Medicine, Tel Aviv University, Tel Aviv 52621, Israel; 3 Sheba Medical Center, The Sheba Talpiot Medical Leadership Program, Tel Hashomer, Ramat Gan 52621, Israel; 4 Department of Pathophysiology, Sechenov First Moscow State Medical University (Sechenov University), 119019 Moscow, Russia; budnik.ivan@gmail.com; 5 The Center for Cancer Research, Sheba Medical Center, Genomics Unit, Tel Hashomer, Ramat Gan 52621, Israel; Ortal.Barel@sheba.health.gov.il (O.B.); yuliakhavkin@gmail.com (Y.K.)

**Keywords:** hemophilia, carrier, X-chromosome inactivation, non-random, mutation, monogenic disease, whole-exome sequencing

## Abstract

Introduction: Hemophilia A (HA) is an X-linked bleeding disorder caused by factor VIII (FVIII) deficiency or dysfunction due to F8 gene mutations. HA carriers are usually asymptomatic because their FVIII levels correspond to approximately half of the concentration found in healthy individuals. However, in rare cases, a carrier may exhibit symptoms of moderate to severe HA primarily due to skewed inactivation of her non-hemophilic X chromosome. Aim: The aim of the study was to investigate X-chromosome inactivation (XCI) patterns in HA carriers, with special emphasis on three karyotypically normal HA carriers presenting with moderate to severe HA phenotype due to skewed XCI, in an attempt to elucidate the molecular mechanism underlying skewed XCI in these symptomatic HA carriers. The study was based on the hypothesis that the presence of a pathogenic mutation on the non-hemophilic X chromosome is the cause of extreme inactivation of that X chromosome. Methods: XCI patterns were studied by PCR analysis of the CAG repeat region in the HUMARA gene. HA carriers that demonstrated skewed XCI were further studied by whole-exome sequencing (WES) followed by X chromosome-targeted bioinformatic analysis. Results: All three HA carriers presenting with the moderate to severe HA phenotype due to skewed XCI were found to carry pathogenic mutations on their non-hemophilic X chromosomes. Patient 1 was diagnosed with a frameshift mutation in the PGK1 gene that was associated with familial XCI skewing in three generations. Patient 2 was diagnosed with a missense mutation in the SYTL4 gene that was associated with familial XCI skewing in two generations. Patient 3 was diagnosed with a nonsense mutation in the NKAP gene that was associated with familial XCI skewing in two generations. Conclusion: Our results indicate that the main reason for skewed XCI in our female HA patients was negative selection against cells with a disadvantage caused by an additional deleterious mutation on the silenced X chromosome, thus complicating the phenotype of a monogenic X-linked disease. Based on our study, we are currently offering the X inactivation test to symptomatic hemophilia carriers and plan to expand this approach to symptomatic carriers of other X-linked diseases, which can be further used in pregnancy planning.

## 1. Introduction

The chromosomal basis of sex determination (i.e., XX in females, XY in males) results in a disparity in gene copy numbers and content between males and females. In humans (and other mammals), the potential imbalance of gene expression from the two X chromosomes in females is corrected by inactivating one X chromosome in the somatic tissues [1]. Beginning in the late blastocyst stage of embryonic development, one of the two X chromosomes is entirely downregulated in each somatic cell, resulting in expression of only one allele at the vast majority of X encoded loci [2]. As a result of X-chromosome inactivation (XCI), heterozygous females are mosaic for X-linked gene expression, with one population of cells expressing genes from the maternal X chromosome and the other population of cells expressing genes from the paternal X chromosome [3]. DNA methylation has been shown to play a major role in XCI. The modulation of gene expression is mediated by the methylation of deoxycytosine residues in the 5’ regions of genes, especially the promoters and GC-rich CpG islands, which are the target areas for methylation [4].

XCI is generally a random process; therefore, female carriers of X-linked recessive diseases are usually asymptomatic in view of the approximately equal expression of both maternal and paternal X chromosome genes. However, on rare occasions, non-random XCI may occur, mainly resulting in the inactivation of the normal X chromosome in a symptomatic female carrier [5,6].

The pattern of XCI may be affected by various factors. Extreme deviation from the expected 50:50 ratio between the two X chromosomes, or XCI skewing, can occur accidentally, but can also be caused by significant genetic processes resulting from selection against cells with X chromosome mutations, which affects cell proliferation [2,7]. Therefore, skewed XCI may be suggestive of a carrier of an X-linked mutation on the inactivated X chromosome.

Hemophilia A (HA) is an X-linked bleeding disorder caused by mutations in the factor VIII gene (F8), located on the long arm of the X-chromosome at Xq28. Female HA carriers are typically asymptomatic because their factor VIII levels correspond to approximately half of the concentration found in healthy individuals, which is generally sufficient for normal hemostasis [8]. However, in rare cases, a carrier may exhibit symptoms of moderate to severe HA due to various reasons, including: homozygosity or compound heterozygosity for F8 gene mutations, Turner syndrome, or abnormally skewed inactivation of the normal X chromosome [9]. In a retrospective, multicenter study of females with moderate to severe hemophilia, skewed XCI accounted for the majority of cases [10].

In this study, we investigated XCI patterns in three karyotypically normal HA carriers presenting with HA phenotype due to skewed XCI. We hypothesized that the molecular mechanism underlying skewed XCI in these symptomatic HA carriers involves a disruptive mutation in a gene located on their non-hemophilic X chromosome, which eventually leads to negative selection and non-random inactivation of that chromosome. Based on our findings, we conducted pregnancy planning for one of the patients, which resulted in a healthy female birth.

## 2. Patients and Methods

### 2.1. Female HA Patients

Three HA carriers exhibiting phenotypic features of severe HA were analyzed for XCI patterns, followed by whole-exome sequencing (WES). The study was approved by the Institutional Review Board of the Sheba Medical Center (Sheba Medical Center Helsinki Committee) on 4 December 2016 (Study number at the Helsinki Committee: SMC-3540-16) and by the Israeli Ministry of Health on 6 July 2017 (Study number at the Ministry of Health: 20172910). All three patients signed an informed consent form.

### 2.2. Genomic DNA Extraction

DNA was isolated from peripheral blood using the Qiagen QIAamp DNA Blood mini QIAcube Kit (Qiagen, Hilden, Germany) according to the manufacturer’s instructions.

### 2.3. XCI Analysis

XCI was studied by PCR analysis of the HUMARA gene [11]. For each DNA sample, two PCR reactions were performed. In one reaction, the template contained DNA digested with HpaII, whereas the other reaction contained undigested genomic DNA. PCR products were subjected to electrophoresis in a sequence analyzer (3500xL Genetic Analyzer, Applied Biosystems, Hitachi), followed by analysis using the GeneMapper software (Applied Biosystems). The degree of skewing was calculated using the equation: (d1/u1)/[(d1/u1) + (d2/u2)], where d1 and d2 represent the digested alleles (+HpaII) of the tested subject and u1 and u2 represent the undigested alleles (-HpaII) [12].

### 2.4. Whole-Exome Sequencing (WES)

DNA samples of patients demonstrating skewed X-chromosome inactivation were examined by WES, followed by X chromosome-focused bioinformatics analysis (Genomics Unit, Center for Cancer Research, Sheba Medical Center).

Exome sequencing was performed using a Sure Select Human All Exon kit V6 on a HiSeq2500 sequencing machine (Illumina, San Diego, CA, USA). For each sample, paired end reads (2 × 150 bp) were obtained and processed. The Illumina Dragen Bio-IT Platform version 3.8 was used to align reads to the human reference genome (hg38) based on the Smith–Waterman algorithm [13], as well as to call variants based on the GATK variant caller version 3.7 [14]. Additional variants were called with Freebayes version 1.2.0 [15]. Variant annotation was performed using KGG-Seq version 1.2 [16]. Further annotation and filtration steps were performed by in-house scripts using various additional datasets.

### 2.5. Confirmation of Mutations and Segregation Studies

Mutations detected by WES were confirmed by PCR and Sanger sequencing, and relevant family members were screened for the respective mutations.

## 3. Results

### 3.1. Patient 1

**Figure 1:**
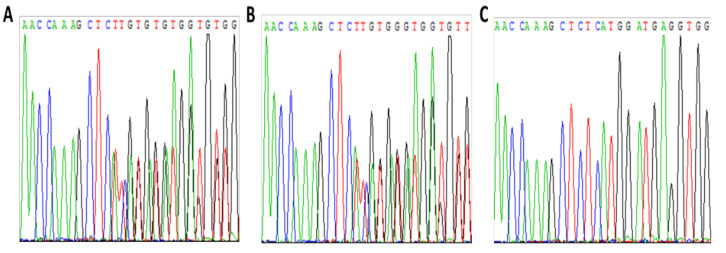
Analysis of patient 1 and her family members for the PGK1 mutation c.1061_1062delCT. (**A**)—patient 1, (**B**)—mother, (**C**)—brother.

Patient 1 is a 35-year-old daughter of a severe HA patient (F8 gene mutation: IVS 5 +2 T > G; splice site mutation) presenting with a severe HA phenotype (FVIII < 1%) due to skewed inactivation of her maternal X chromosome. X chromosome-targeted WES analysis demonstrated that she is a heterozygous carrier of a frameshift mutation (NM_000291.4; c.1061_1062delCT; p.A354fs*4) in the phosphoglycerate kinase 1 (PGK1) gene (Figure 1A) encoding phosphoglycerate kinase 1, which is involved in the glycolysis pathway.

**Figure 2:**
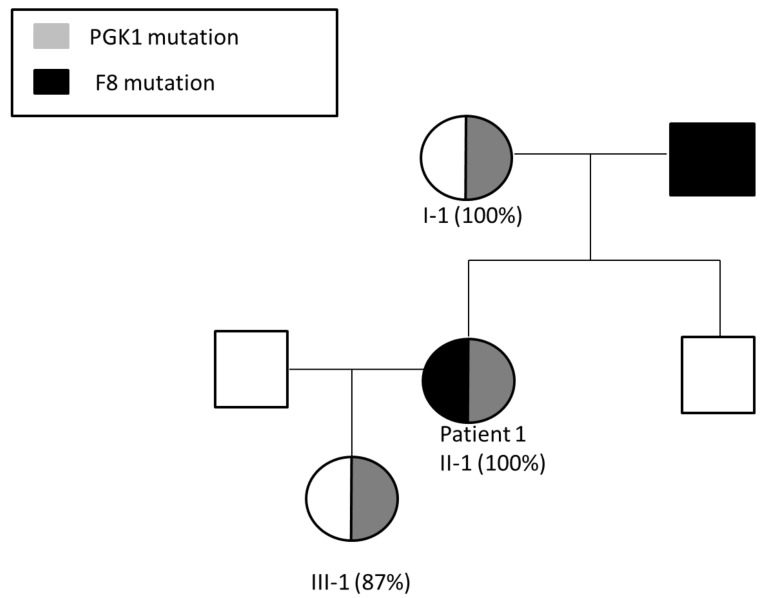
Segregation of F8 and PGK1 mutations in the pedigree of patient 1, demonstrating three generations of skewed inactivation of the X-chromosome bearing the mutant PGK1 gene (%XCI is indicated in parentheses). Patient 1 (II-1), her mother (I-1) and her daughter (III-1) demonstrate skewed inactivation of the same X chromosome. Patient 1 presents with HA phenotype due to a F8 mutation inherited from her father.

Segregation studies including the patient’s parents and brother showed that her mother is a heterozygous carrier of the same mutation in PGK1 (Figure 1B). Further analysis of XCI demonstrated skewed inactivation of the X chromosome bearing the mutant PGK1 gene in the patient’s mother as well (Figure 2). The patient’s brother inherited the normal PGK1 gene from their mother (Figure 1C).

#### Pregnancy Planning for Patient 1 

Following our analysis, patient 1 decided to undergo in vitro fertilization (IVF) combined with pre-implantation genetic diagnosis (PGD) in order to reduce the chances of giving birth to a male fetus with either the F8 gene mutation or the PGK1 gene mutation. She received comprehensive genetic counseling, and chose the option of a female embryo carrying the mutant PGK1 gene (but not the mutant F8 gene), based on the assumption that her daughter is highly likely to inactivate the X chromosome bearing the mutant PGK1 gene. The patient’s daughter was subsequently examined for X-chromosome inactivation, and demonstrated 87% inactivation of her maternal X chromosome bearing the mutant PGK1 gene (Figure 2).

Patient 1 (II-1), her mother (I-1) and daughter (III-1), who are carriers of the PGK1 mutation, all demonstrate skewed inactivation of the X-chromosome bearing the mutant PGK1 gene. Patient 1 presents with severe hemophilia A due to being a carrier of a F8 mutation inherited from her father in addition to the PGK1 mutation inherited from her mother.

### 3.2. Patient 2

**Figure 3:**
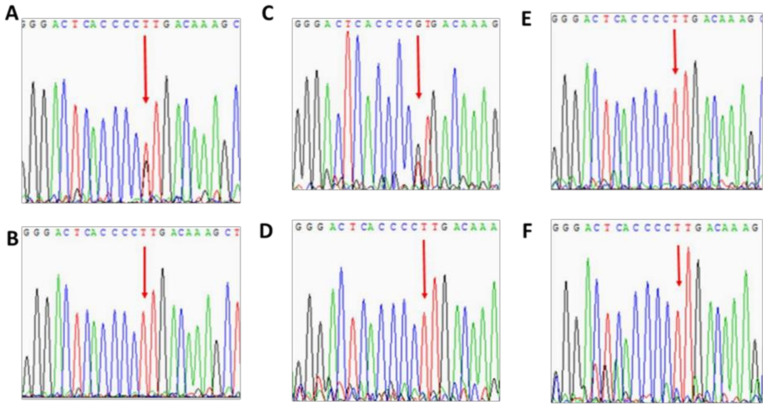
Analysis of family members of patient 2 for the SYTL4 mutation c.1655A > C. (**A**)—patient 2, (**B**)—father, (**C**)—mother, (**D**)—sister 1, (**E**)—sister 2, (**F**)—brother.

**Figure 4:**
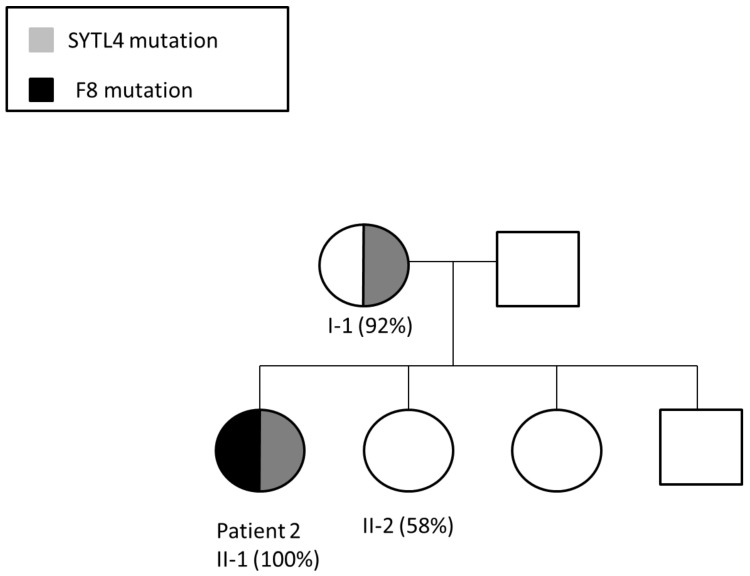
Segregation of F8 and SYTL4 mutations in the pedigree of patient 2, demonstrating two generations of skewed inactivation of the X chromosome bearing the mutant SYTL4 gene (%XCI is indicated in parentheses). Both patient 2 (II-1) and her mother (I-1) demonstrate skewed inactivation of the same X chromosome. Patient 2 presents with HA phenotype due to de novo F8 intron 22 inversion, which occurred on her paternal X-chromosome and skewed inactivation of the X chromosome bearing the mutant SYTL4 gene inherited from her mother. The patient’s son inherited the X chromosome bearing the F8 intron 22 inversion.

Patient 2 is a 32-year-old female HA patient (FVIII 3%; heterozygous for F8 intron 22 inversion) with no family history of HA. Her HA phenotype results from a sporadic mutation on her paternal X chromosome, combined with skewed inactivation of her maternal X chromosome. X chromosome-targeted WES analysis identified a missense mutation in the synaptotagmin-like protein 4 (SYTL4) gene (NM_001129896; c.1655A > C; p.K552T) encoding the SYTL4 protein, which is involved in intracellular membrane trafficking via interaction with Rab GTPases. Based on segregation studies, patient 2 (Figure 3A) inherited the mutant SYTL4 gene from her mother (Figure 3C), who also demonstrates skewed XCI (Figure 4). Both her sisters and her brother received the X chromosome bearing a normal copy of the SYTL4 gene (Figure 3D–F). Both sisters demonstrate normal random patterns of XCI (Figure 4).

Patient 2 chose spontaneous pregnancy with neither PGD nor prenatal diagnosis, eventually giving birth to a boy with severe HA.

### 3.3. Patient 3

**Figure 5:**
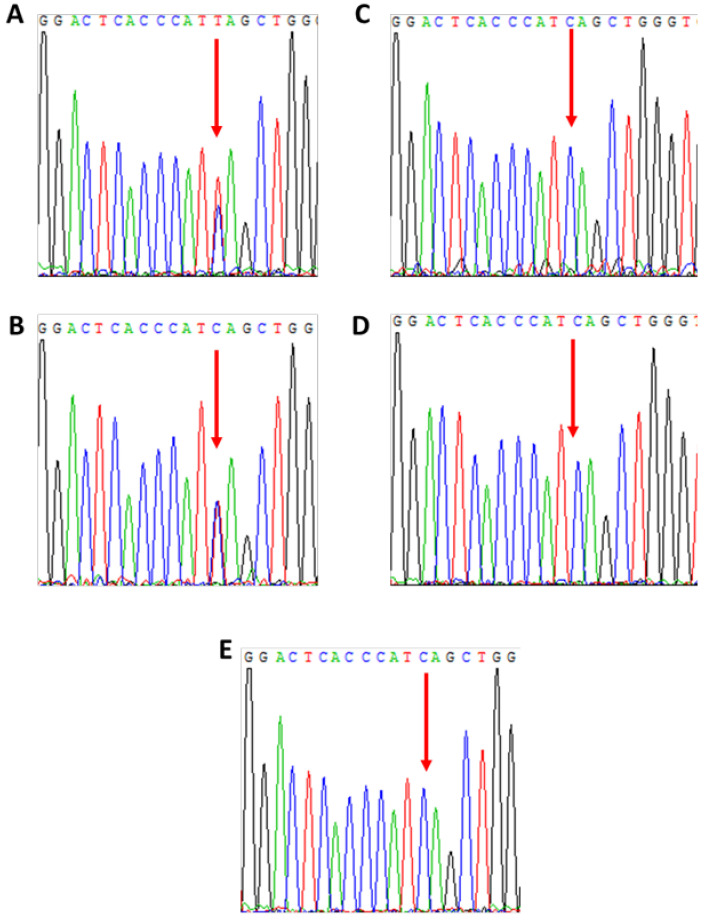
Analysis of family members of patient 3 for the NKAP mutation c.175 C> T. (**A**)—patient 3, (**B**)—mother, (**C**)—sister, (**D**)—brother 1, (**E**)—brother 2.

**Figure 6:**
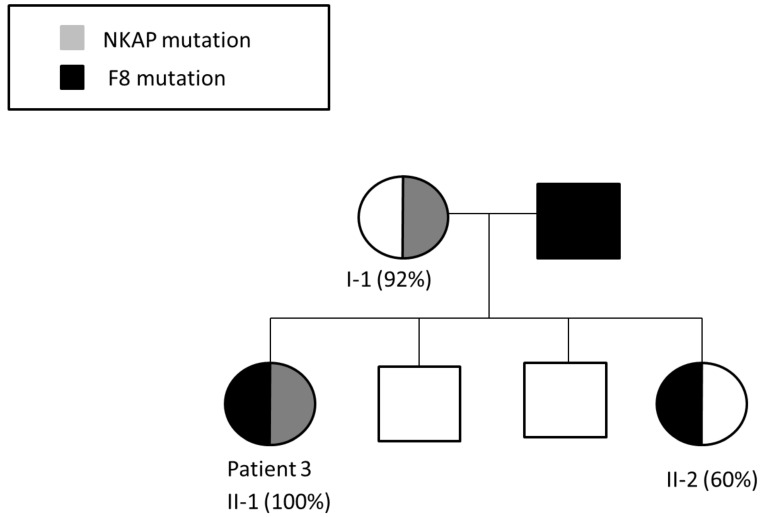
Segregation of F8 and NKAP mutations in the pedigree of patient 3, demonstrating two generations of skewed inactivation of the X-chromosome bearing the mutant NKAP gene (%XCI is indicated in parentheses). Patient 3 and her sister are both obligatory HA carriers carrying the F8 intron 22 inversion mutation. Patient 3 also carries the NKAP mutation and demonstrates complete inactivation of the X chromosome bearing the NKAP mutation, thus presenting with the HA phenotype. The patient’s sister demonstrates random XCI and is an asymptomatic HA carrier. Their mother, who is a carrier of the NKAP mutation, demonstrates skewed XCI.

Patient 3 is a 44-year-old daughter of a severe HA patient presenting with HA phenotype (FVIII 4%; heterozygous for F8 intron 22 inversion) due to skewed inactivation of her maternal X chromosome. X chromosome-targeted WES analysis identified a nonsense mutation in the NF-kappa B activating protein (NKAP) gene (NM_024528; c.175C > T; p. Q59*) encoding the NKAP protein, which is involved in activation of the ubiquitous transcription factor NF-Kappa B. Based on segregation studies, patient 3 (Figure 5A) inherited the mutant NKAP gene from her mother (Figure 5B), who also demonstrates skewed XCI (Figure 6). None of the patient’s siblings (two healthy brothers and one sister) carry the mutant NKAP gene (Figure 5C–E). The patient’s sister, who is an obligatory carrier of HA, has normal phenotype in terms of FVIII level and a normal random XCI pattern (Figure 6).

## 4. Discussion

Many reasons for extreme XCI have been proposed in the literature. It may occur by chance because of a small cell number. It may also be caused by mutations in the XIST gene, the gene responsible for the inactivation process [17], although such mutations are rare and do not explain most of the familial cases [18]. There are a few studies of monozygotic female twins with extreme XCI patterns that suggest that skewed XCI may be a result of the twinning process itself [19,20]. Another potential mechanism that possibly explains familial cases of skewed XCI is selection against cells with a disadvantage caused by a mutation on the silenced X chromosome [21].

One of the X-linked disorders commonly associated with skewed XCI is X-linked mental retardation (XLMR). The involvement of X-linked genes in mental retardation is probably related to the higher expression of X-linked genes in the central nervous system compared with autosomal genes [22], and is supported by a higher incidence of mental retardation in males compared to females. Indeed, potential causative mutations for XLMR have been identified in several X-chromosomal genes [23]. Skewed XCI has been reported in about half of the families with XLMR, suggesting that XLMR mutations represent a group of X-linked mutations characterized by a general defect in cell viability or proliferation [24]. Skewed XCI has been demonstrated to play a role in the development of symptomatic phenotypes in female carriers of Duchenne and Becker dystrophinopathies [25], Wiskott–Aldrich Syndrome [26], G6PD [27], and other X-linked disorders. Karyotypic abnormalities (deletions, duplications, X chromosome-autosomal translocations) have been implicated in preferential inactivation of the abnormal X chromosome in some cases of skewed X-chromosome inactivation [28].

Garagiola et al. [29] have recently reported that low FVIII activity in HA carriers is associated with skewed XCI. Of note, analysis of XCI patterns in our cohort of HA carriers at the Israeli National Hemophilia Center revealed no evidence of significant correlation between XCI patterns and FVIII levels (data not shown), which is in agreement with the lack of correlation between XCI patterns and FVIII levels in HA carriers reported by Orstavik [30].

In this study, we focused our attention on the investigation of the mechanism of skewed XCI in three female HA patients in an attempt to explain the reasons for this phenomenon. We found that all three of them exhibited deleterious mutations on the inactivated X chromosome, associated with familial XCI skewing. Patient 1 carries a heterozygous frameshift mutation in the PGK1 gene. PGK1 plays an essential role during glycolysis, generating ATP by catalyzing the conversion of 1,3- diphosphoglycerate to 3-phosphoglycerate [31,32]. PGK1 is a ubiquitous enzyme expressed in all somatic cells [33]. PGK1 was reported to affect three types of tissue: red blood cells, central nervous system and muscles. Variable symptoms have been observed in patients with PGK1 deficiency, including chronic anemia, exercise intolerant myopathy, muscle weakness, cramping, myalgia, myoglobinuria, and intellectual disability [34]. Segregation studies conducted in the family of patient 1 support significant involvement of the PGK1 frameshift mutation in X chromosome silencing, eventually leading to severe HA in our patient, who is also an obligatory HA carrier.

Patient 1 has sought our assistance with pregnancy planning in order to avoid the risk of giving birth to a male child affected with either hemophilia A or PGK1 deficiency. To the best of our knowledge, no pregnancy planning has been ever conducted for a hemophilia A carrier with XCI skewing due to a deleterious mutation on the inactivated X chromosome. The patient received comprehensive genetic counseling, which was based on the evidence of familial silencing of the X chromosome bearing the mutant PGK1 gene (observed in both patient 1 and her mother; Figure 2), and on the assumption that this X chromosome is highly likely to undergo silencing in further generations of females carrying it. Based on our advice, patient 1 chose the option of a female embryo carrying the mutant PGK1 gene (but not the mutant F8 gene). Indeed, the newborn female demonstrated 87% inactivation of her maternal X chromosome bearing the mutant PGK1 gene (Figure 2). Thus, our WES findings, combined with segregation studies, guided us throughout the process of successful family planning for patient 1.

Patient 2 carries a heterozygous missense mutation in the SYTL4 gene. The SYTL4 gene, also referred to as granuphilin, directly interacts with members of the RAB family, genes known to be involved in autism disorders [35]. SYTL4 also directly interacts with three other genes associated with autism: STX1A, SNAP25 and STXBP1. [35] This gene is important in neuronal system development and is implicated in neurological and psychological diseases [35,36]. Targeted null/knockout mice mutant in SYTL4 phenotypes showed abnormal behavior and neurological disorders [36]. Of note, the mother of patient 2, the only female relative carrying the SYTL4 missense mutation, also exhibits skewed XCI, in contrast to the patient’s sisters who exhibit neither the SYTL4 mutation nor skewed XCI. This finding strongly supports our notion that the SYTL4 mutation plays a key role in skewed inactivation of our patient’s non-hemophilic X-chromosome, resulting in her HA phenotype.

Patient 3 carries a nonsense mutation in the NKAP gene encoding the NKAP protein. NKAP is a conserved protein comprised of 415 amino acids, and is assumed to play an important role in hematopoiesis. Males with NKAP germline missense mutations exhibit developmental delay, hypotonia, joint contractures, behavioral abnormalities, Marfanoid habitus, and scoliosis [37]. NKAP deficiency reduces hematopoiesis of stem cells and increases their apoptosis. Pajerowski et al. showed that conditional knockout of NKAP in mice results in perinatal lethality, with pups dying 1–3 days after birth, and exhibiting a dramatic hematopoiesis blockade [38]. It should be noted that both patient 3 and her sister are obligatory HA carriers. However, the patient’s sister, who does not carry the NKAP mutation, exhibits a typical HA carrier phenotype with balanced XCI. In contrast, the patient’s mother who carries the NKAP mutation, demonstrates skewed XCI, thus strongly supporting the significant association between the NKAP mutation and skewed XCI.

Our findings suggest that the major reason for skewed X inactivation in our female HA patients is selection against cells with a disadvantage caused by a deleterious mutation on the silenced X chromosome. This concept is strongly supported by the fact that all female relatives of the three patients who carry the same mutations (three generations in the family of patient 1, two generations in the families of patient 2 and patient 3) exhibit skewed XCI patterns involving the same X chromosome.

A recent study by Janczar et al. [39] of 18 female hemophilia patients concluded that every case of female hemophilia warrants wide genomic analysis, since this may reveal other morbidities beyond hemophilia. In agreement with this conclusion, and based on our study, we are currently offering whole-exome sequencing to symptomatic hemophilia carriers, which can be further used in pregnancy planning, based on the aforementioned considerations implemented in pregnancy planning for patient 1. [40] Furthermore, we plan to expand this approach to symptomatic carriers of other monogenic X-linked diseases.

## Data Availability

The data presented in this study are available on request from the corresponding author.
